# Prevalence of anxiety disorders: a population-based epidemiological study in metropolitan area of Casablanca, Morocco

**DOI:** 10.1186/1744-859X-6-6

**Published:** 2007-02-10

**Authors:** Nadia Kadri, Mohamed Agoub, Samir El Gnaoui, Soumia Berrada, Driss Moussaoui

**Affiliations:** 1 Ibn Rochd University psychiatric center, Rue Tarik Ibn Ziad, 20000 Casablanca, Morocco

## Abstract

**Background:**

In Morocco, no epidemiological study has been conducted to show the current prevalence of mental disorders in the general population. The aim of the present study was to assess the prevalence and comorbidity of anxiety disorders in Moroccan subjects.

**Methods:**

We used cross-sectional study, with a representative sample of Casablanca city. Direct interviews used the Mini International Neurpsychiatric Interview in its validated Moroccan Arabic version

**Results:**

Among 800 subjects, 25.5% met criteria of at least one current anxiety disorder: Panic Disorder (2%), Agoraphobia (7.6%) Social phobia (3.4), Obsessive Compulsive Disorder (6.1%), Post Traumatic Stress Disorder (3.4%), Generalized Anxiety Disorder (4.3%)

**Conclusion:**

The results are generally similar to those of Western countries. Future studies need to replicate these results and to concentrate on their impact on the quality of life and the cost of such conditions in the community.

## Background

Recent epidemiological studies of anxiety disorders provided evidence of their high frequency in the general population worldwide [1]. In the United States of America, the recent National Comorbidity Survey Replication (NCS-R) found a lifetime prevalence rate of 28.8% [2] and a twelve-month prevalence of 18.1% [3].

These disorders are mostly chronic, and have a negative impact on the life of patients and they can impair severely the daily functioning of the people suffering from them.

They also have a high comorbidity between various anxiety disorders and with other mental disorders: depression, alcohol/substance dependence and abuse, suicide [4].

On the other hand, anxiety disorders appear to be more common in community populations than in clinical settings [5].

In Morocco, in spite of the high number of patients seen in our daily practice, so far no epidemiological study has been conducted to show the current prevalence of these disorders.

The objectives of the current study were to obtain:

- Data on prevalence of anxiety disorders in Moroccan subjects residing in metropolitan area of Casablanca, Morocco, in a population-based study.

- Prevalence and nature of comorbidity of these disorders with other mental disorders.

## Methods

The study was cross-sectional one. Men and women, 15 years old or above, were randomly selected using systematic sampling from eight prefectorats of Casablanca, Morocco. The sample was stratified according the gender and the prefectorats. Streets, blocks and then households were randomly selected. For each chosen family one member was randomly selected. The interviews took place, in the houses of the interviewers "face to face". It was stated from the beginning that in case of refusal the person will not be replaced.

For the sampling the authors used the most recent national census (1994). Sample size was calculated assuming in the population prevalence of these disorders is 20.1% (ECA study) with 4% variability. The calculated sample size at 99% confidence level was 666.

The study protocol and questionnaires were reviewed and approved by ethics committee, faculty of Medicine, University of Casablanca.

Participants gave their verbal informed consent before entering in the study.

The standardized instrument which used was the Mini International Neuro-psychiatric Interview (M.I.N.I) in its validated Moroccan Arabic language [6] according to the definitions and criteria of DSM IV [7]. Definition of current prevalence was one month for panic disorder, agoraphobia, social anxiety, obsessive compulsive disorder and post traumatic stress disorder and 6 months for generalized anxiety disorder. Used MINI explores life time prevalence only for panic disorder and agoraphobia.

A questionnaire available from the authors inquiring about socio-demographic data was also used.

Qualified and trained medical doctors assisted in filling up the questionnaires. The training consisted on five theoretical sessions on anxiety disorders according DSMIV criteria and MINI (two hours each), then Four sessions on inter rater reliability test.

The pilot study was conducted in 20 subjects aiming adapting the questionnaire and identifying the difficulties on the field.

Data analysis was performed on PC microcomputer using Epi info in its sixth version.

### Statistical analysis

This study was a descriptive one. The data were analysed using the 6^th ^version of the Epi Info software (CDC Atlanta). Statistical methods used univariate analysis; authors described sociodemographic characteristics and contingency tables of each disorder. Analysis of variance (ANOVA) and t test were used for group comparisons. Chi-square and Fisher's exact test were used for analysis of categorical data. Level of significance was set at 0.05 for all analysis.

## Results

Out of 874 approached people, 800 had completed the files; 400 men and 400 women. These 74 people refused to participate to the study. The reported causes were lack of time, no interest about the study and no specified cause.

### Demography and habits

The mean age of the population was 32.2 years (SD = 12.8), ranging from 15 to 80 years. Single people represented 58% of the population; 35.4% of them were married.

The level of unemployment was 24.1%; 41.5% had a professional activity. The remaining people were students or housewives.

Concerning the level of education, 15.02% had no education, 15.8% had a primary school level (1–5 years), 52.6% had 6 to 13 years of education and 15.3% had a university level.

For the monthly income (1US Dollar is equivalent to about 10 Dh), 18% had less than 1000 Dh per month, 36.4% had 1000–2000 Dh, 33.5% had 2000–5000 Dh, 9.1% had 5000–10000 Dh and 3% had more than 10.000 Dh per month.

For alcohol and substance use/dependence, 20.4% were current tobacco users and 13.3% were dependent. 3.9% were cannabis users and 1.8% were dependent. 5.1% were alcohol users and 2.3% were dependent.

### A) Panic Disorder (PD)

The lifetime prevalence of the Panic Disorder was 2.3%; meanwhile the current prevalence was 2%. This current PD was associated to agoraphobia in 56.3% (n = 9), and without agoraphobia in 43.7% (n = 7). Limited symptoms of current panic disorders were found in 0.9% of cases (n = 7).

The epidemiological characteristics of the people with Current panic Disorder were as follows: female in 87.5% of cases (n = 14), the mean age was 29.4 years (SD = 10.9), with 62.5% of single and age of onset of 21.8 years (SD = 6.9). Comorbidity with social phobia was shown in 25% of cases, with specific phobia in 50%, with obsessive compulsive disorder in 37.5%, with PTSD in 18.8%, and with Major depressive Disorder in 18.8%.

### B) Agoraphobia

For the agoraphobia, the lifetime prevalence was 8.4%. The current one was 7.6%, with association to panic disorder in 14.7%. In 1.6% this agoraphobia had antecedent of PD.

The epidemiological characteristics of people with current Agoraphobia were as follows: female in 90.2% (n = 50) of cases, with a mean age of 30.5 years (SD = 12.9) [15–60 years]. Marital status was shared between single (57.4%) and Married (34.4%) ones, unemployed people in 70.5% of cases. Age of onset was 18.5 years (SD = 12.3) and comorbidity was seen with Social phobia (19.7%), Specific phobia (60.7%), PTSD (9.8%), OCD (34.4%), and current major depressive disorder (MDD) in 16.4%

### C) Social phobia

In this sample, 3.4% reached the full criteria of social phobia. Most of them were female 82.8% (n = 24). The mean age was 30.5 years (SD = 12.1), 72.4% of then were single, 65.5% had no professional activity. The age of onset was 15.1 years (SD = 5.8). This disorder was comorbide to: a current MDD in 20.7%, Panic disorder in 13.8%, Agoraphobia in 41.4%, Specific Phobia in 55.2%, OCD in 34.5%, PTSD in 13.8%, and dependence to nicotine in 10.3%, to alcohol in 3.4%, to Substance in 6.9%

Lifetime prevalence was not explored because the MINI life time does not inquire about it

### D) Obsessive Compulsive Disorder

The prevalence of OCD was 6.1%. Most of the people were female 93.8%. The mean Age was 33.7 years (SD = 12.2), 45.8% of them were single and 41.7% married, 64.6% had no professional activity, and the age of onset was 25.4 years (SD = 11.8). In this sample, 29% had obsessions and compulsions, 20.8% reported obsessions only and 50% reported Compulsions only. The most frequent Types of obsessions were religious, contamination and aggressive ones. The most commonly occurring compulsions included cleaning and washing, checking and repeating compulsions.

Comorbidity was seen with: Panic disorder in 12.5%, Agoraphobia in 43.8%, Social Phobia in 20.8%, Specific Phobia in 64.6%, PTSD in 8.3%, current MDD in 16.7% and dependence of nicotine in 2.1%

### E) Post Traumatic Stress Disorder

In this sample 12.1% reported have been exposed to traumatic events. These events were mostly drowning, fire, traffic accident and rape. Meanwhile, the prevalence of PTSD was 3.4 %. Third of the sample (66.6%) was female with a mean age of 34.1 years (SD = 13.1), 48.1% were single and 33.3% married, 51.9% had no professional activity, the mean Age of onset was 26.6 years (SD = 12.2). This disorder was comorbid with: Panic Disorder in 11.1%, Agoraphobia in 22.2%, Social Phobia in 14.8%, Specific Phobia in 33.3%, OCD in 14.8%, current MDD in 7.4%, Dependence of nicotine in 18.5% and alcohol abuse in 3.7%

### F) Generalized Anxiety Disorder

The prevalence of GAD was 4.3%. In 91.1% people suffering from it were female, the mean age was 35.1 years (SD = 13.1), 43.5% were single, 40.2% married and 67.4% had no professional activity.

## Discussion

This study is the first one conducted in Moroccan and Maghrebian population in community sample exploring the prevalence of anxiety disorders, and it's the first time we have data in this field which are, in general in accordance with the literature except for the prevalence of Obsessive compulsive disorders which was higher in this sample with higher prevalence among women.

Among 800 subjects, 25.5% met criteria of at least one current anxiety disorder: Panic Disorder (2.3%), Agoraphobia (7.6%) Social phobia (3.4), Obsessive Compulsive Disorder (6.1%), Post Traumatic Stress Disorder (3.4%), Generalized Anxiety Disorder (4.3%). We found a high Comorbidity between anxiety disorders and major depressive disorder.

In this study the current prevalence of anxiety disorder was 25.5% which is in accordance with the literature [3,8]. However The European Study of the Epidemiology of Mental Disorders projects found a 12 month prevalence of any anxiety disorder 6.4% which is lower than previous studies and the current one [9]. In Arab region, Okasha and Ashour [10] found the same socio-demographic pattern than the current study with higher risk for young women and single people to suffer from anxiety disorders.

According to literature, lifetime prevalence of PD was 2.3% and the current one was 2% with a higher prevalence for women, younger and single subjects and a high comorbidity with other anxiety disorders and major depressive disorders [11-14].

It was associated to agoraphobia in about a half of cases. During recent years, several epidemiological studies conducted around the world determined the relative consistency of the prevalence of panic disorder in the community. The annual prevalence is about 1% [11], and the lifetime prevalence has been found to range between 1.4% and 3.5% [11-13], with lower rates in some Asian countries. Rates were found higher in women than in men, in younger, and widowed, separated and divorced subjects. Comorbidity of panic disorder with other anxiety disorders and affective disorders has also been consistently reported in these studies.

In Arab countries, Weissman et al. [14] in the Cross-national epidemiology study of panic disorder in 10 countries, including over 40000 subjects, found that lifetime prevalence rates for panic disorder ranged from 1.4% to 2.9%. Mean age at first onset was usually in early to middle adulthood. The rates were higher in females than in male subjects in all countries. Panic disorder was associated with an increased risk of agoraphobia and major depression in all countries.

On the other hand, Weissman et al. [14] found a predominance of females in the one-month and 6-month prevalence rates of panic disorder in most countries. A comorbidity pattern with agoraphobia is observed in 29.5%-58.2% of subject with panic disorder [11-13].

Current prevalence of social phobia was 3.4%, predominantly in women, single, and middle-aged people. Mean age was older than for other anxiety disorders, and the age of onset was in the adolescence. A high comorbidity was seen with MDD, other anxiety disorders and alcohol and substance dependency [11,13,15,16]. In the literature, various epidemiological studies have indicated social anxiety disorder is a frequent condition in the community. The lifetime prevalence rates ranges from 2% to 4% for the most severe forms and up to 10% or even 15% meanwhile the National comorbidity survey (NCS) found 12 months rates of 7.9% [11,13,16].

In our study, the lifetime prevalence of OCD was 6.1%. Female gender was predominant. Both obsessions and compulsions were seen in about one third of cases. Compulsions only were found in half of them. In ECA study, 1-month prevalence was 1.3%, 6-month prevalence 1.5% and lifetime prevalence was 2.5% [17]. We have no explanation for this higher lifetime prevalence of OCD as well as for the predominance of female subjects, to be confirmed by others studies. But we can hypothesis that in Morocco the religious obsessions are more frequent in Morocco following the example of cultures with more religious connotation such as Turkey, Israel, Bahrain Egypt and Saudi Arabia. On the other hand, biological factor might also be an explanation

A study conducted by Okasha et al. [18] found that the prevalence rate of OCD, in a psychiatric outpatient Egyptian study was 2.3%, meanwhile the same authors found a highest prevalence of obsessive compulsive symptoms (62.4%) in a sample of Egyptian psychiatric patients [19]. These two studies were the only studies in the region found to be compared to our, however they were conducted in clinic settings no in community.

In our study, 12.1% of the sample was exposed to traumatic events at least once during their lives, 3.4% of them satisfied PTSD diagnosis. Female gender was predominant, and comorbidity with all types of anxiety and with depression was relatively high. This Estimates of the lifetime prevalence of PTSD from surveys of the general adult population ranged from 1% to 12.3% [20,21].

Estimates of the lifetime prevalence of PTSD from surveys of the general adult population ranged from 1% to 12.3%. Using data from two sites in the Epidemiologic Catchment Area program, Helzer et al. [20] reported a lifetime prevalence of 1%.

Evidence suggests that exposure to potentially traumatic events may be more common that once thought, and that risk factors for PTSD include personal and biographical histories at the time of exposure to the extreme event, characteristics of the event itself, and characteristics of the post-exposure environment. For example, Resnick et al. [21] examined PTSD prevalence rates associated with different types of extreme events in a nationally representative community sample of women. The overall prevalence was 12.3% lifetime. This prevalence varied by type of traumatic event. Women who reported interpersonal violence were more likely to meet criteria for lifetime (25.8%) and current (9.4%) PTSD than women who reported other stressors only.

The current prevalence of GAD was 4.3% with predominance of female, without professional activity. For the NCS, the current prevalence was 1.6 and a 12-month prevalence was of 3.1 %, and GAD was more common in women, and had a very high lifetime comorbidity of 90% with a wide spectrum of other psychiatric disorders [22].

We noted some limitations of the present study:

- Small size of the sample which limited only in the city of Casablanca. The next step is the explore the anxiety disorders in a National randomised sample

- Socio-cultural factors might be explanation for some differences such as the prevalence of OCD;

## Conclusion

For the first time in Morocco, systematic epidemiological data on anxiety disorders are available. The results are generally similar to those of Western countries. Future studies need to concentrate on their impact on the quality of life and the cost of such conditions in the community.

## Competing interests

The author(s) declare that they have no competing interests.

## Authors' contributions

NK and AM conceived and coordinated the study. NK, MA, SE and SB designed and performed questionnaire and carried out the data collection. NK, MA and DM drafted the manuscript. All authors read and approved the final manuscript.

**Table 1:** Characteristics of the study sample

	N	%
**Gender**		
Male	400	50
**Age (years)**		
15–29	385	48.1
30–44	274	34.2
45–59	114	14.2
≥ 60	27	3.4
**Marital status**		
Single	464	58.0
Married	283	35.3
Divorced	26	3.2
Widowed	27	3.4
**Educational level**		
Illiterates	121	15.2
Primary school	131	16.3
Secondary school	425	53.1
Graduated	123	15.3
**Profession**		
Remunerated Employment	332	41.5
Unemployed	68	58.5
**Prevalence**		
Panic Disorder	18	2.3
Agoraphobia	61	7.6
Social Phobia	29	3.6
Specific Phobia	114	14.3
Obsessive-compulsive Disorder	49	6.1
Post-traumatic-stress Disorder	27	3.4
Generalized Anxiety Disorder	34	4.3

**Table 2:** Comorbidities of anxiety disorders

	PD	Agoraphobia	Social phobia	OCD	PTSD
PD	-	14.7	13.8	12.5	11.1
Agoraphobia	56.3	-	41.4	43.8	22.2
Social phobia	25	19.7	-	20.8	14.8
OCD	37.5	34.4	34.5	-	14.8
PTSD	18.8	9.8	13.8	8.3	-
MDD	18.8	16.4	20.7	16.7	7.4

PD: panic disorder, OCD: obsessive compulsive disorder, PTSD: post-traumatic disorder, MDD: major depressive disorder
